# Light-Cured Junction Formation and Broad-Band Imaging Application in Thermally Mismatched van der Waals Heterointerface

**DOI:** 10.3390/ma17163988

**Published:** 2024-08-11

**Authors:** Liyuan Cheng, Qinglin Quan, Liang Hu

**Affiliations:** Key Laboratory of Novel Materials for Sensor of Zhejiang Province, College of Materials and Environmental Engineering, Hangzhou Dianzi University, Hangzhou 310018, China; liyuancheng115@163.com (L.C.); quanqinglin2022@163.com (Q.Q.)

**Keywords:** transition metal chalcogenophosphates, black phosphorus, van der Waals heterostructure, thermal mismatch, interface, broad-band imaging

## Abstract

Van der Waals (vdW) heterostructures are mainly fabricated by a classic dry transfer procedure, but the interface quality is often subject to the vdW gap, residual strains, and defect species. The realization of interface fusion and repair holds significant implications for the modulation of multiple photoelectric conversion processes. In this work, we propose a thermally mismatched strategy to trigger broad-band and high-speed photodetection performance based on a type-I heterostructure composed of black phosphorus (BP) and FePS_3_ (FPS) nanoflakes. The BP acts as photothermal source to promote interface fusion when large optical power is adopted. The regulation of optical power enables the device from pyroelectric (PE) and/or alternating current photovoltaic (AC–PV) mode to a mixed photovoltaic (PV)/photothermoelectric (PTE)/PE mode. The fused heterostructure device presents an extended detection range (405~980 nm) for the FPS. The maximum responsivity and detectivity are 329.86 mA/W and 6.95 × 10^10^ Jones, respectively, and the corresponding external quantum efficiency (EQE) approaches ~100%. Thanks to these thermally-related photoelectric conversion mechanism, the response and decay time constants of device are as fast as 290 μs and 265 μs, respectively, superior to current all FPS-based photodetectors. The robust environmental durability also renders itself as a high-speed and broad-band imaging sensor.

## 1. Introduction

Currently, the demand for high-performance, small-sized sensors has driven the development of devices toward lightweight and miniaturized forms. Two-dimensional (2D) materials with atomic-level thickness, characterized by a wide range of types and heterostructure construction ways without considering lattice mismatch, provide diverse avenues for studying various physical effects and designing multifunctional optoelectronic devices [1]. However, the fabrication of high-quality 2D heterointerfaces still poses significant challenges due to Fermi level pinning, strain effects, molecular adsorption, and surface/interface defects [2]. Extensive efforts have been dedicated to optimizing the heterointerface environment through techniques such as thermal treatment [3], laser irradiation [4], and incorporation of thin dielectric layers [5]. These approaches enable effective van der Waals (vdW) gap engineering as a crucial methodology for designing high-performance devices.

According to the difference of working principle, 2D photodetectors can be classified into photon-based and thermal-effect-based ones [6]. In general, constructing heterostructures is the most common technical strategy to improve the performance shortcomings of individual materials [1]. However, the investigation of most heterostructures only focuses on band engineering optimization, and the generation, conversion, and efficient utilization of thermal effects have not received sufficient attention. Among them, the photothermoelectric effect (PTE) can effectively collect photo-generated thermal carriers, enabling more efficient energy harvesting and offering a promising choice for high-efficiency optoelectronic energy conversion and photodetection [7]. Therefore, it has become a distinctive class among many emerging detector types and is widely used in phototransistor [8,9], high-speed [10,11,12,13,14], wideband [15,16,17,18,19], and high-gain [20] photodetection. So far, there have been a few research studies that can simultaneously reveal and modulate the thermal effect and photovoltaic (PV) effect in traditional heterostructure. Therefore, it is highly necessary to design a vdW photodetector that is compatible with both band engineering and thermal effect studies.

Transition metal chalcogenophosphates (TMCPs) are a highly regarded class of 2D layered materials that exhibit rich optoelectronic [21,22,23,24,25,26], magnetic [27,28,29,30,31,32,33,34], and catalytic properties [35,36,37,38,39,40]. Their general chemical formula can be written as *M*P*X*_3_ (*M* represents transition metals such as Fe, Ni, Mn, etc.; P represents phosphorus; and *X* represents sulfur, selenium, or tellurium). Among numerous *M*P*X*_3_ compounds, FePS_3_ (FPS) stands out for its adjustable spectral response range (1.32~3.40 eV) [41], abundant magnetic structures [27,28,29,30,42], and excellent environmental stability [43], which contribute to its high application potential in the field of photodetection [44,45,46,47,48]. FPS has an indirect bandgap structure, a low hole carrier mobility [44,45], and a long transit time for photo-generated charge carriers between electrodes. As a result, pure FPS often exhibits low photo gain [43,44,48]. Building heterojunctions can improve response speed [46] and expand the response frequency range [45], but millisecond-level speeds are not compatible with high-speed applications, and there is still room for further improvement in the response waveband. Meanwhile, there is relatively limited research on the thermal effect management of FPS. Exploring how to utilize the thermal effects to enhance the aforementioned characteristics has become a new approach to promote the practical application of FPS.

As an efficient thermoelectric semiconductor [49], black phosphorus (BP) holds promise in addressing the thermal insensitivity issue of FPS. Moreover, due to its tunable direct bandgap [50,51], it can be utilized in the design of heterojunction combinations with band bending configurations, laying the foundation for the construction of FPS-based detectors with broad spectral response, high sensitivity, and fast response. In this work, a unique thermally-mismatched BP−–FPS vdW heterostructure is proposed, where the difference in photothermal property between FPS and BP is utilized to control the vdW gap. This enables the device from pyroelectric (PE) and/or alternating current photovoltaic (AC–PV) mode to mixed PV/PTE/PE mode. The optimized device exhibits a wide spectral response ranging from near-ultraviolet to near-infrared wavelengths (405–980 nm), with significantly improved response speed reaching the microsecond level, and an internal quantum efficiency approaching 100%. By acquiring Raman and photocurrent imaging data within the same heterojunction region, the thermal stress and photocurrent distribution at the heterointerface is visualized, providing essential evidence for understanding the origin of the aforementioned high performance. The above research demonstrates that high-quality interface optimization and thermal effect management are of crucial guidance for enhancing the overall performance of detectors. Furthermore, the outstanding environmental stability after interface fusion also contributes to stable and high-resolution imaging applications of the device.

## 2. Materials and Methods

### 2.1. Synthesis of Heterostructure

The FPS, BP, and graphite bulk crystals (purity above 99.99%) were purchased from SixCarbon Technology (Shenzhen, China). According to standard dry transfer procedure, these single crystals were first mechanically exfoliated as nanoflakes by blue adhesive plastic films (SPV-224R, Nitto, Osaka, Japan), and then those small and thin ones were attached onto the polydimethylsiloxane (PDMS) stamp (DC184, Dow Corning, Midland City, TX, USA) for next-stage stacking. Through a simple thermal release process (85 °C, 10 min), the chosen nanoflakes can be stacked in sequence to form a multilayer heterostructure by a micro-transfer system (E1-T, METATEST, Nanjing, China).

### 2.2. Device Fabrication and Measurements

The Cr (5 nm)/Au (45 nm) electrodes were defined onto Si/SiO_2_ substrates by a patterned photolithography process. Prior to usage, the substrates with surface oxidizing layer thickness of 300 nm were fully washed with ethanol (AR, Aladdin, Shanghai, China) and acetone (AR, Aladdin, Shanghai, China) and finally treated in oxygen plasma for removing residual organics. In order to realize the prototype photoelectric device, the heterostructure was fabricated on pre-patterned Cr/Au electrodes without capping *h*-BN to examine the working stability of devices. Specifically, two electrode terminals were first covered by BP and few-layer graphite nanoflakes, respectively. And then the FPS flake was put onto the above two flakes to bridge them. The whole procedure was implemented in an argon-filled glove box for protecting samples. A detailed preparation flow chart can be found in Supplementary Figure S1. Previous work has shown that the contact resistance of FPS and Au can be significantly improved by using few-layer graphite materials as contact electrodes [46]. The photoelectric measurements were conducted on a probe station coupled with optical fibers with different wavelengths (405, 520, and 980 nm). The initial incident optical powers are set as *P*_405_ = 0.74 mW/cm^2^, *P*_520_ = 0.45 mW/cm^2^, and *P*_980_ = 1.18 mW/cm^2^, respectively, which can ensure the linearity of *I*–*V* curves. When the powers are elevated tenfold, the *I*–*V* characteristics start to evolve into the nonlinear feature, which can be regarded as the onset of interface fusion. This conversion can be accurately verified in optical power-dependent photocurrent experiments (Figure S2). A temperature control chamber (CH400-190-V, GoGo Instruments, Shanghai, China) was employed for the adjustment of ambient temperature and the study of light-sensitive pyroelectric properties. The electrical data were recorded in a high-precision semiconductor analyzer (Keithley 2602B, Tektronix, Beaverton, OR, USA).

### 2.3. Characterizations

The quality of single crystals was characterized by the X-ray diffraction (XRD, SmartLab, Rigaku, Tokyo, Japan) method. The morphological images of exfoliated nanoflakes and stacked heterostructures were captured by an optical microscope (BX53M, Olympus, Tokyo, Japan). The fingerprint scattering peaks (single point scan) and thermal strain distribution (mapping scan) of the heterostructure region were confirmed by a Raman spectrometer (ScanPro Advance, METATEST, Nanjing, China) equipped with an excitation light source with a wavelength of 532 nm. The photocurrent mapping data were collected from a photocurrent scanning measurement system (MStarter 200, METATEST, Nanjing, China). An atomic force microscope (AFM, NanoWizard 4-NanoScience, JPK Instruments, Berlin, Germany) and its Kelvin probe mode (KPFM) were used to determine sample thickness and the contact potential difference between the sample surface and the Pt/Ir probe (PPPEFM, NanoSensors, Neuchatel, Switzerland) tip. The photothermic data were recorded by a thermal imaging camera (H13, HIKVISION, Hangzhou, China).

## 3. Results and Discussion

**Figure 1:**
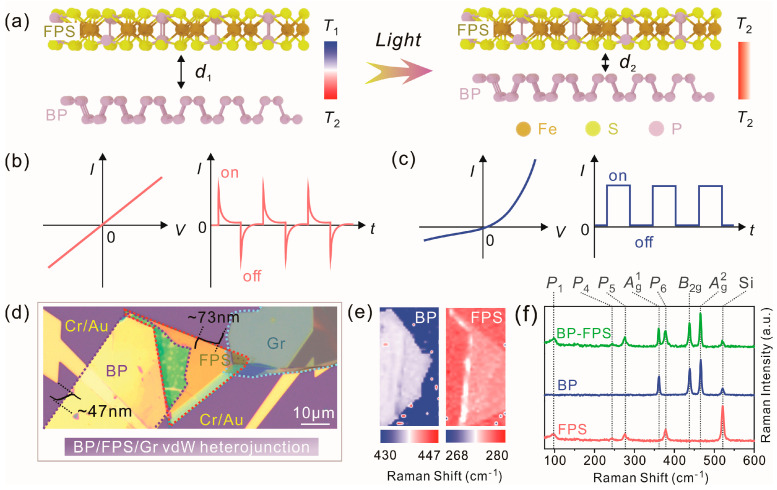
(**a**) Schematic diagram of light-induced interface fusion for BP−–FPS heterostructure. (**b**,**c**) Representative light sensitive *I*–*V* and *I*–*t* curves before and after interface fusion. (**d**) Optical microscopic image from BP/FPS/Gr vdW heterostructure device. The corresponding height profiles of BP and FPS nanoflakes are shown around. The purple, red, and blue dotted lines denote the BP, FPS, and Gr regions, respectively. (**e**) Raman peak mapping of BP and FPS components from the *B*_2g_ mode in BP and the *P*_5_ mode in FPS, respectively. (**f**) Raman spectral comparison for different regions.

In general, the contact of vdW heterostructure can be improved through a moderate baking process, for instance, 220 °C for 2 h [3]. Considering that BP is thermally instable, we utilized optical means to fuse the interface and optimize the contact. A similar method has been reported to improve magnetic heterostructure interfaces by laser shocking engineering [4]. The schematic diagram of the BP−–FPS stacked heterostructure and the corresponding optical microscopic image are shown in Figure 1a,d, respectively. Here, the vdW gap *d*_1_ and *d*_2_ are defined as the differences between the interlayer spacings and the covalent bond lengths [2]. In general, *d*_1_ is often above 0.5 nm and sometimes even exceeds several nanometers, which mainly depends on the types and the contents of inserted molecules [5]. To ensure electron transport across the FPS−–BP interface, the *d*_1_ gap should be controlled below 1 nm for finite electric fields. And *d*_2_ is the distance that can enhance interlayer interactions, which is often ideally regarded around 0.23 nm (the vdW gap value of an Ar atomic dimer [2]). For heterointerfaces, the *d*_2_ values might be larger and are typically below 0.5 nm in experiments [4]. When two materials stay closer to each other, in addition to normal junction effect, complex interface environments, e.g., residual strains, traps, and/or defect species, might also influence the transport properties. According to the double logarithmic plotting for *I*–*V* curves (Figure S3, discussed later), the ohmic-like transport mechanism plays a major role in the conduction process at this stage. In this regard, a linear *I*–*V* characteristic can be expected, as displayed in Figure 1b. The presence of the vdW gap, residual strains, and interface defects hinder the diffusion of majority carriers, and the spatial charge region is hard to form. Compared with bulk BP with centro-symmetric *mmm* structure, the centro-symmetry is broken in micromechanically-thinned BP nanoflakes and shows an *m* spatial group when it is in a monolayer form. Therefore, the pyroelectric (PE) effect can be expected in thinned BP [49]. The photothermally-induced temperature fluctuation results in the inversion of polarized electric potentials [52], which enables the observable peak photocurrents in the current versus time (*I*–*t*) curve.

When the incident optical power increases, the out-of-plane temperature difference can fuse the BP−–FPS interface and further reduce the gap to *d*_2_ for the formation of a spatial charge region. After the heterostructure is thermally cured by high-power incident light, the *I*–*V* characteristics will change into the nonlinear feature (Figure 1c) and obey the spatial charge limited conduction (SCLC) behavior (Figure S3). The photocurrent is also significantly enhanced with the optical power above a few mW/cm^2^ (Figure S2). A similar phenomenon has also been reported in BP−–InSe heterojunction [53]. From the Raman peak mapping (Figure 1e), the majority of thermal stress in the heterostructure region has been relaxed by an adjacent FPS layer; there are obvious Raman redshifts for BP and FPS components located at the left edge of the heterostructure overlapped area, indicating that these interfacial atoms possibly still experience thermal tensile stress. Accompanied with the formation of a spatial charge region, the PE mechanism is gradually replaced by the photovoltaic (PV) effect in the photoelectric conversion process. As depicted in Figure 1c, the *I*–*V* curve appears sub-linearity, and there are photocurrent plateaus in *I*–*t* cycling tests.

The XRD patterns of bulk single crystals are displayed in Figure S4. All the diffraction peaks exactly correspond to the crystalline planes of FPS and BP, indicating their good crystallization. The as-prepared vdW heterojunction was comprised by a BP nanoflake, a FPS nanoflake, and a graphite nanoflake with thickness of ~47 nm, ~73 nm, and ~12 nm, respectively (Figure S5). In Figure 1d, their contour lines are marked with purple (BP), red (FPS), and azure (Gr) dotted lines for better observation. For the region with thermal stress relaxation, microscopic Raman spectroscopy was also used to reveal the phonon vibration information of the heterostructure. The characteristic Raman peaks in individual FPS, BP, and BP−–FPS heterostructure are marked in the upper of Figure 1f. The peaks of 361.6, 438.8, and 466.3 cm^−1^ represent the interlayer $A_{g}^{1}$ mode (out-of-plane direction), the intralayer $B_{2g}$ mode (in-plane zigzag direction), and the intralayer $A_{g}^{2}$ mode (in-plane armchair direction), respectively, which are consistent with the previous results of BP Raman research [50,51]. For the FPS region, it includes four observable phonon modes, labeled *P*_1_ (99.1 cm^−1^), *P*_4_ (245.3 cm^−1^), *P*_5_ (275.7 cm^−1^), and *P*_6_ (377.8 cm^−1^). The *P*_1_ mode is related to the vibration of Fe atoms, the *P*_5_ mode involves the in-plane *E*_g_ vibration of P_2_S_6_ clusters, and *P*_4_ and *P*_6_ correspond to out-of-plane *A*_1g_ vibration of P_2_S_6_ clusters [29]. The peak positions of every component almost remain unchanged after the formation of the heterojunction, indicating that no stress exists in the sampling point.

**Figure 2:**
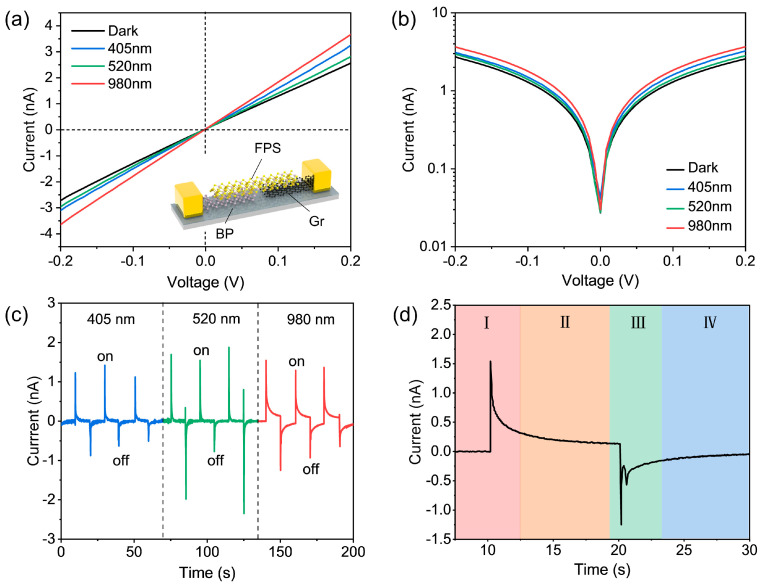
(**a**,**b**) *I*–*V* characteristic curves in linear (**a**) and logarithm (**b**) scales under dark and illumination states with different incident light wavelengths of 405, 520, and 980 nm. Inset in (**a**) is the 3D model illustration of the BP−–FPS−–Gr device. (**c**) Temporal photocurrent excited by different light sources. (**d**) Enlarged view for photocurrent versus time (*I*–*t*) curve under illumination of 980 nm. A +2 V bias voltage is applied to the device.

To reveal the initial performance of the BP−–FPS−–Gr heterostructure device, we implemented incident light wavelength-dependent photodetection measurements at ambient condition. By adopting three typical wavelengths (incident power: *P*_405_ = 0.74 mW/cm^2^, *P*_520_ = 0.45 mW/cm^2^, and *P*_980_ = 1.18 mW/cm^2^), the broadband detection capability and the response kinetic behavior of the device are systematically investigated. The diameters of all laser beams are controlled around 55 μm for fully covering the overlapped area (255.52 μm^2^, see Figure S6), and the focusing position is kept at the same position. As shown in Figure 2a, the measured *I*–*V* characteristics display broadband photoresponse from near ultraviolet (405 nm) to near-infrared band (980 nm). All the curves exhibit perfect linearity within a scanning bias range from −0.2 V to +0.2 V and strictly cross the origin. Such features not only suggest good ohmic contacts between materials and Cr/Au electrodes but also demonstrate that the heterojunction between BP and FPS is not formed. Symmetric *I*–*V* characteristics plotted in logarithmic scale (Figure 2b) further support the above viewpoint. Figure 2c depicts a group of wavelength-dependent temporal photocurrent under *V* = +0.2 V. The spike-like *I*–*t* curves coincide with the characters of pyroelectric (PE) and/or alternating current photovoltaic (AC–PV) effect [54,55,56], i.e., switching on the laser corresponds to an upward peak current, and turning off the laser leads to a downward peak current. It is noteworthy that, in addition to the spike currents, the photocurrent generated by 980 nm illumination possesses a small and stable current plateau, which is distinct from the case of 405 nm and 520 nm.

To understand the above process, the current variation (980 nm) at each on/off cycle is divided into four stages in Figure 2d. When the device is illuminated by the laser, the temperature of the BP component within the heterostructure rapidly rises (see photothermic data in Figure S7). The fluctuation in temperature further regulates the spontaneous polarization in BP and thus causes the transient release of polarized charges. Phenomenally, a considerable d*T*/d*t* > 0 leads to the generation of pyroelectric signals, i.e., achieving an instantaneous rise of the output current (*I*_PE_∝d*T*/d*t*). As d*T*/d*t* ≈ 0 (*I*_PE_ ≈ 0), the second stage displays a small current plateau when irradiation is maintained. This can be explained by the normal photoconductivity (*I*_PC_) from BP because the photon energy of incident light is lower than the bandgap of FPS. In the third stage, when the laser illumination is suddenly turned off, the temperature in BP rapidly drops, resulting in the opposite distribution of thermal charges in BP, and finally the output current has a reversed spike feature. The appearance of a bimodal distribution means that at least two pyroelectric processes participate in this stage. We can speculate this process as follows. The first *I*_PE_ peak is normally linked with the temperature fluctuations of the BP component. As for the second one, due to a broken inversion symmetry in BP−–FPS interfaces, the in-plane self-polarization might also be formed, thereby contributing to the second *I*_PE_ peak. A similar polarization phenomenon has been also reported in the BP–WSe_2_ heterostructure [3]. In the fourth stage, the temperature drops to room temperature and remains stable, so the thermopotential disappears again and the output current recovers to the dark-state level.

It is worth noting that the maximal on/off ratio is achieved under 980 nm illumination rather than 405 nm and 520 nm. Considering that the photon energy of 980 nm closely aligns with the resonant absorption of BP and falls below that of FPS, the generated photocurrents at 980 nm primarily consist of two components: pyroelectric currents (*I*_PE_) resulting from instantaneous temperature changes in BP and intrinsic photoconductive currents (*I*_PC_) originating from BP itself. In contrast, for the photocurrents at 405 nm and 520 nm, the majority of photons are absorbed by FPS counterparts. Due to its indirect band structure, *I*_PC_ becomes negligible and enslaved to a less efficient photoelectric conversion process in FPS, thereby rendering only observable *I*_PE_ peaks and small on/off ratios.

**Figure 3:**
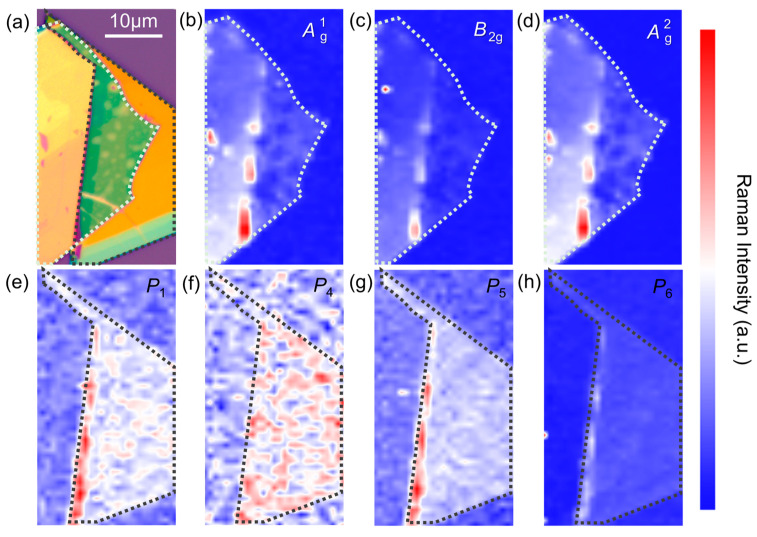
(**a**) Optical microscopic image of selected Raman mapping region. (**b**–**d**) Raman intensity mapping images from BP modes. (**e**–**h**) Raman intensity mapping images from FPS modes. The white and black dotted lines represent the BP and FPS regions, respectively.

We further studied the microscopic origin of the pyroelectric phenomenon. The mapping of the Raman peak position can reveal the spatial distribution of thermal strain (Figure 1e), while the mapping of intensity can provide the information about the magnitude distribution of thermal strain (Figure 3). As the scanning region determined in Figure 3a, we collected the corresponding intensity images (Figure 3b–h) from different modes in BP and FPS. For the BP region, the maximal values in intensity correspond to the magnitude distribution of thermal strain, which is mainly located at the left edge of the heterojunction region. Considering that a part of the BP nanoflake is underneath the FPS nanoflake, the capping of FPS would influence the photothermal absorption of BP especially for 405 nm and 520 nm laser beam. Therefore, the temperature variation at the border line between the covered and uncovered BP regions contributes to pyroelectric phenomenon. The FPS also exhibits a certain inhomogeneity in intensity distribution. The similar thermal strain distribution in FPS and BP at the border line suggests that there is significant interlayer coupling. This demonstrates that the pyroelectricity might originate from a synergistic role between BP and FPS.

**Figure 4:**
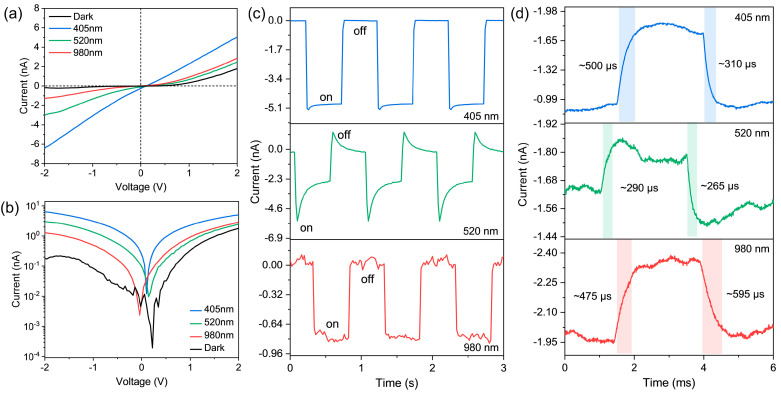
(**a**,**b**) *I*–*V* characteristic curves in linear (**a**) and logarithm (**b**) scales under dark and illumination with incident light wavelengths of 405, 520, and 980 nm. (**c**,**d**) Cycled photoresponse measurements (**c**) and temporal photocurrent (**d**) illuminated by different light sources. The device is driven under −2 V bias voltage.

Back to the scenario depicted in Figure 1c, as the incident optical power increases (*P*_405_ = 7.42 mW/cm^2^, *P*_520_ = 4.56 mW/cm^2^, and *P*_980_ = 11.8 mW/cm^2^), the *I*–*V* curves in Figure 4a show good asymmetric photodiode characteristics under both dark and illumination conditions. The difference is that the curves in dark, 520 nm, and 980 nm illuminations exhibit obvious rectification transport characteristics, indicating that the Schottky barriers impede the movement of thermal/photogenerated carriers. However, with an increase in incident light energies (405 nm), photogenerated carriers overcome these barriers and result in quasi-ohmic *I*–*V* behavior. This character is consistent with the analysis of type-I band alignment (discuss later). The dark current *I*_dark_ at −0.2 V decreases from −2.723 nA (before interfacial fusion) to −0.012 nA (after interfacial fusion). A 230-fold reduction in *I*_dark_ and optically modulated open-circuit voltage (Figure 4b) fully demonstrate the effective formation of the junction region. By using different chopping frequencies (2 Hz for Figure 4c; 201 Hz for Figure 4d), all the photocurrents appear repeated on/off features. The adjacent waveform maintains good consistency, indicating the stability of device operation. The only difference is that the pyroelectric peaks obviously appear under 520 nm laser irradiation, and other excitation conditions only give rise to typical square wave signals. We can qualitatively understand this phenomenon from two aspects. First, the 405 nm and 980 nm illuminations are well above the intrinsic absorption of FPS and BP components, respectively. Irradiation-induced interfacial fusion weakens the influence of temperature fluctuation (d*T*/d*t*) on the pyroelectric process and thus improves the thermal conductivity. The formation of spatial charge regions boosts the separation of photogenerated carriers and gives rise to stable plateaus photocurrents. Second, for the incident wavelength of 520 nm, in addition to the normal band-edge absorption of FPS (plateaus photocurrent), there are a portion of thermal carriers generated by the interband absorption between BP and FPS. The residual thermal strain in the BP−–FPS interface regulates spontaneous polarization, concurrently contributing to the pyroelectric current *I*_PE_.

We next discuss the conduction mechanism before and after interface fusion. Double logarithmic relations of *I*–*V* curves for both conditions have been plotted in Figure S3. From the fitting, it is found that the slopes before interface fusion are close to 1, indicating ohmic-like conduction mechanism (*I*~*V*^α^, *α* = 1) in a pristine heterostructure device. As interfaces fuse, the plots become nonlinear and the conductions obey the trap-filled-limited mechanism (TFL, *I*~*V*^α^, 1 < *α* < 2) and Child’s law (*I*~*V*^α^, *α* ≈ 2) [57]. Such a spatial-charge-limited conduction (SCLC) behavior demonstrates the presence of abundant interface traps, for example, surface oxidized BP species. When the high-power illumination is involved, interface traps start to participate in the conduction process, thereby SCLC mechanism dominating over the other processes.

As shown in Figure 4d, the response time constant (*τ*_rise_: photocurrent increases from 10% to 90% of the final value) and the decay time constant (*τ*_decay_: photocurrent decreases from 90% to 10% of the initial value) of the BP−–FPS−–Gr device are determined to be 500 μs and 310 μs for 405 nm illumination, 290 μs and 265 μs for 520 nm illumination, and 475 μs and 595 μs for 980 nm illumination, respectively. To the best of our knowledge, the response speeds shown here are better than those of other FPS-based vdW photodetectors [44,45,46,47,48]. According to the definition of responsivity *R* = (*I*_photo_ − *I*_dark_)/*P*_in_A [45], where *P*_in_ is the incident light power density and A is the effective illumination area (255.52 μm^2^), the *R* values of the device under −2 V bias are calculated as ~329.86 mA/W (405 nm), ~242.88 mA/W (520 nm), and ~36.58 mA/W (980 nm), respectively. Likewise, the specific detectivity (D^*^) and external quantum efficiency (EQE) are estimated to be 6.95 × 10^10^ Jones and 100.99% for 405 nm, 5.12 × 10^10^ Jones and 57.92% for 520 nm, and 7.71 × 10^10^ Jones and 4.63% for 980 nm by the expressions D* = *R*A^1/2^/(2e*I*_dark_)^1/2^ and EQE = hc*R*/e*λ* [58], where e, h, c, and *λ* are the elementary charge, the Planck constant, the velocity of light, and the wavelength of incident light, respectively. It is worth noting that the performances of FPS in the heterostructure at 405 nm and 520 nm gain considerable improvement, and the quantum efficiency almost approaches unity at 405 nm. Such highly efficient energy conversion together with extended detection waveband to 980 nm render the combination of FPS and BP as a promising strategy to advance the application of 2D FPS material.

**Figure 5:**
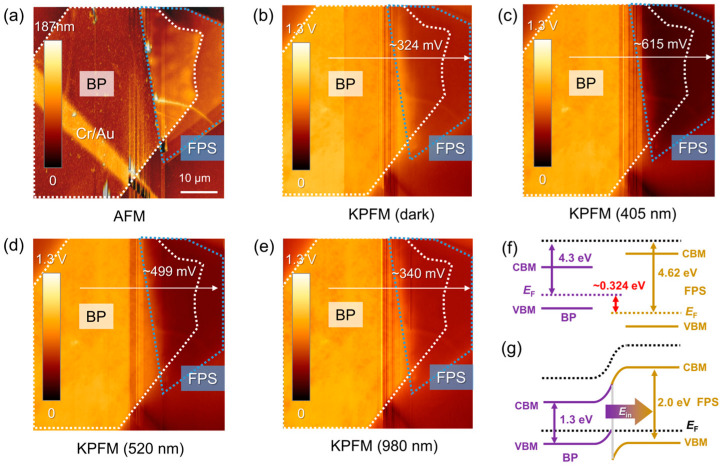
(**a**–**e**) AFM height image (**a**) and corresponding KPFM images collected under dark condition (**b**) and different illumination wavelengths of 405 nm (**c**), 520 nm (**d**), and 980 nm (**e**). The contact potential differences between BP and FPS region are shown in images. The white and grey dotted lines are the regions of BP and FPS, respectively. (**f**,**g**) Band alignments before and after BP−–FPS contact.

In order to reveal the band alignment between FPS and BP, we conducted KPFM scanning measurements. Figure 5a is the AFM height picture, and Figure 5b–e are incident wavelength-dependent surface potential distribution images recorded at the same region. The contact potential difference (CPD) between the AFM tip and the sample surface can be expressed by *V*_CPD_ = (*Φ*_tip_ − *Φ*_sam_)/e, where *Φ* is the work function. For the difference in *Φ* between BP and FPS, Δ*Φ* = *Φ*_BP_ − *Φ*_FPS_ = e[*V*_CPD@BP_ − *V*_CPD@FPS_] = e·Δ*V*_CPD_. According to the profiles of electric potentials (Figure S8), the Δ*V*_CPD_ under dark condition is around 324 mV, thereby Δ*Φ*~0.324 eV can be achieved. Furthermore, the Fermi levels of BP and FPS can be estimated to be −4.30 eV and −4.62 eV, respectively, since *Φ*_tip_ is around 4.5 eV. The optical bandgaps (*E*_g_) of two kinds of nanoflakes are determined as 1.29 eV (BP) and 1.99 eV (FPS) by the Tauc plot fittings for absorption spectra (Figure S9). The band alignment and the bending diagram are shown in Figure 5f,g. Taking the staggered way of energy levels into consideration, the BP−–FPS heterojunction can be regarded as type-I band structure with a built-in electric field (*E*_in_) from BP to FPS. Δ*V*_CPD_ varies from 615 mV (405 nm) and 499 mV (520 nm) to 340 mV (980 nm) and shows a consistent variation trend with *R*, D*, and EQE. Since the Δ*V*_CPD_ between FPS and Gr is negligible (Figure S10), we can conclude that the major contribution of photocurrent comes from the BP−–FPS heterojunction.

**Figure 6:**
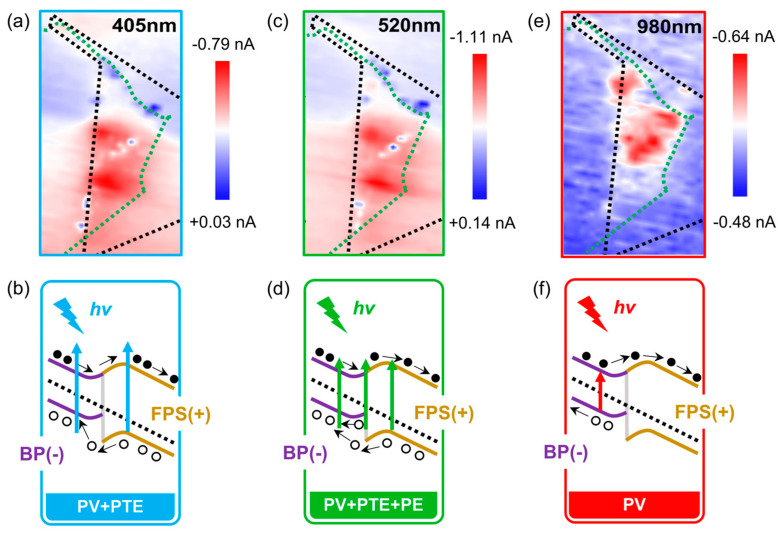
(**a**,**c**,**e**) Scanning photocurrent mapping of vdW heterostructure device working at −0.2 V under illumination of 405 nm (**a**), 520 nm (**c**), and 980 nm (**e**). The green and black dotted lines are the regions of BP and FPS, respectively. (**b**,**d**,**f**) Corresponding energy band bending diagrams, spectral absorption, and possible photodetection mechanisms.

In addition to the pyroelectric phenomena associated with BP, it is crucial to consider the possibility of photothermoelectric (PTE) effect at BP−–FPS interfaces due to the expected temperature difference in this heterojunction pair. As shown in Figure 6a,c,e, we concurrently collected photocurrent mapping data under three typical incident laser. For 405 nm and 520 nm, there is obvious indication about the presence of opposite photocurrents (i.e., blue spot-like regions). They correspond to the opposite potential gradient Δ*V*_PTE_ = (*S*_BP_ − *S*_FPS_)(*T*_BP_ − *T*_FPS_) with the *E*_in_ direction (Figure 6b,d,f) [59,60], where *S* is the Seebeck coefficient and *T* is the temperature. When the device is negatively biased, *I*_PTE_ positively contributes to the photovoltaic current *I*_PV_ (red spot-like regions) and together improves the photo gain. Similar spatial distributions of *I*_PV_ and *I*_PTE_ at 405 nm and 520 nm suggest similar photoelectric conversion processes (Figure 6b,d). Therefore, the spatial mapping of photocurrents can provide a clear guidance to trace potential PTE effect.

To sum up, the absorption/response of 980 nm (1.27 eV) is mainly ascribed to the BP nanoflake, while the absorption/response of 405 nm (3.06 eV) and 520 nm (2.38 eV) are attributed to the synergetic effect of FPS and BP nanoflakes. In detail, the photon energy of 980 nm coincides with the optical bandgap (1.29 eV) of BP and cannot be absorbed by FPS so that almost all photocurrents are localized within the BP layer. Due to the lack of visual PTE evidence in Figure 6e, the PV effect can be considered as the main response mechanism. The photon energy of 520 nm corresponds to the ideal resonant absorption of FPS (Figure S9) and can also be slightly absorbed by the BP flake. The superposition of peak currents in *I*–*t* curves indicates the possible contribution of PE and/or AC–PV effects. Therefore, the response of 520 nm illumination originates from the “PV + PTE + PE” synergetic effect in the FPS−–BP heterostructure. For 405 nm, only square wave response signals appear in *I*–*t* curves, which indicates that the PE and/or AC–PV mechanism can be ignorable. In this regard, the “PV + PTE” synergetic effect becomes the main response mechanism. The incident optical power largely influences the working mode of the device. When the device is illuminated by low-power laser, the photoresponse is mainly the PE and/or AC–PV signal; however, the response would transform to the mixed PV/PTE/PE mode under the high-power case. In order to demonstrate this evolution, we implemented power-dependent tests (Figure S2). It is found that the photocurrents gradually become more linear when the power is set above levels of several mW/cm^2^, which might correspond to the interface fusion of BP−–FPS heterojunction. The incorporation of the PV and PTE processes can promote the photoelectric conversion efficiency. In contrast, a sublinear deviation at low-power illuminations reveals a poor conversion process, i.e., light-driven PE process.

**Figure 7:**
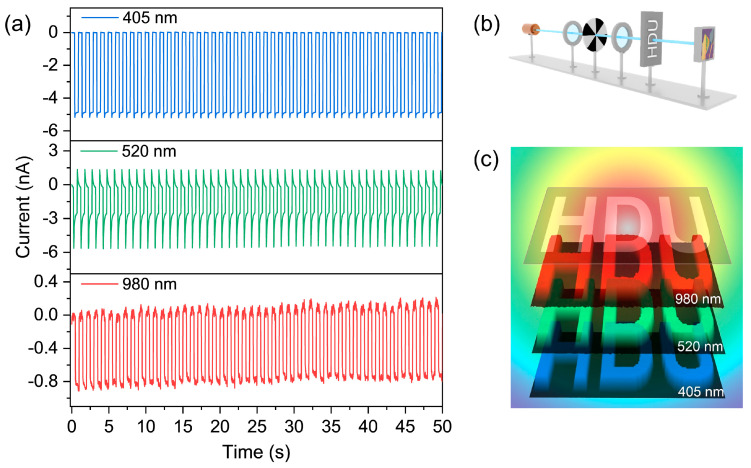
(**a**) Durability tests of device under different illumination conditions. (**b**) Single-pixel photodetection apparatus. (**c**) Broad-band imaging photos using customized “HDU” mask.

Finally, the working stability of a BP−–FPS−–Gr heterojunction photodetector is shown in Figure 7a. After 50 on/off cycles, the photocurrents and dark currents both maintain high consistency, indicating good device reliability and repeatability. In the ambient condition without additional protection, the main detection performance remains almost unchanged for three months. The stability demonstrated here is expected to be associated with the type-I heterostructure, wherein the lone pair electrons of BP can undergo transfer to FPS, thereby impeding their susceptibility to oxidation in an oxygen- and water-rich environment. Thanks to the extended detection waveband and microsecond-level response speed, the device exhibits a high-speed imaging capability from near violet to near-infrared regions. Through an integrated electrically-controlled X–Y displacement platform to move “HDU” mask (Figure 7b), the single-pixel imaging effect can be clearly distinguished (Figure 7c), highlighting the potential of high-speed and broadband imaging application.

It is worth noting that the FPS material exhibits an Ising-type antiferromagnetic behavior with a Neel temperature of approximately 118 K [29]. At room temperature, it undergoes a transition to the paramagnetic state without disturbing the generation, dissipation, and transport processes of excitons in heterojunctions. Therefore, the contribution of the magnetic structure of FPS does not account for the photodetection performance achieved in this study. It is anticipated that by modulating the coupling between the antiferromagnetic order and excitons below the phase transition temperature, intriguing magneto-photodetection phenomena might be observed. Ongoing investigations are being conducted on this aspect.

## 4. Conclusions

In summary, a thermally-mismatched BP−–FPS−–Gr vdW heterostructure is established on pre-patterned Cr/Au electrodes. The vdW gap, residual strains, and defect species in BP−–FPS interfaces hinder the formation of an effective spatial charge region, enabling an ohmic-like photoconduction behavior. Due to the PE effect in thinned BP, the photoresponse exhibits a spike-like current characteristic, which is closely correlated with broken symmetry induced self-polarization in BP. Raman mapping results provide a spatial tool to visualize the origin of the PE effect. The AC–PV effect might also be responsible for this phenomenon. As the optical power increases to a few mW/cm^2^, the heterointerfaces are fused, which is beneficial for the formation of a spatial charge region in type-I heterojunction and the occurrence of multiple photoelectric conversion processes. The trap-filled-limited mechanism plays a major role at this stage. By means of a wavelength-dependent KPFM and scanning photocurrent technique, a mixed photoelectric conversion mechanism including PE, PV, and PTE is proposed to understand the emerging phenomenon. The involvement of BP extends the response range of FPS from 405 nm to 980 nm. Specifically, the response of 980 nm is mainly ascribed to the BP component, while the responses of 405 nm and 520 nm are attributed to the synergetic effect of FPS and BP nanoflakes. The achieved maximal *R*, D*, and EQE are 329.86 mA/W, 6.95 × 10^10^ Jones, and 100.99%, respectively. The most rapid response and decay time constants are 290 μs and 265 μs, respectively. Based on the above merits, the heterostructure device can steadily work for three months and display broad-band imaging capability without additional protection. This work demonstrates that high-quality interface optimization and thermal effect management are of crucial guidance for enhancing the overall performance of detectors.

## Data Availability

Data are contained within the article and Supplementary Materials.

## Supplementary Materials

The following supporting information can be downloaded at: https://www.mdpi.com/article/10.3390/ma17163988/s1, Figure S1: Preparation flow chart of BP−–FPS−–Gr vdW heterostructure; Figure S2: Incident-power-dependent photocurrents generated by 405, 520, and 980 nm laser excitation; Figure S3: Double logarithmic plot fitting of *I*–*V* characteristics before and after interface fusion; Figure S4: Digital photographs and indexed XRD patterns of FPS and BP single crystals; Figure S5: AFM height profiles of micromechanically BP, FPS, and graphite nanoflakes; Figure S6: The diameter of the laser beam and the overlapped area of BP−–FPS heterostructure; Figure S7: Photothermal imaging pictures of Si/SiO_2_ and Si/SiO_2_−–BP−–FPS substrates before and after laser illumination; Figure S8: KPFM line-scan profiles of BP−–FPS region under dark condition, 405 nm, 520 nm, and 980 nm laser irradiation; Figure S9: Absorption spectra and corresponding Tauc plots for determining optical bandgap of BP and FPS nanoflakes; Figure S10: KPFM mapping images for the FPS−–Gr heterostructure region under dark and different wavelength excitation conditions.
