# Supplementary material for: Light-Cured Junction Formation and Broad-Band Imaging Application in Thermally Mismatched van der Waals Heterointerface
Source: Materials (Basel). 2024 Aug 11;17(16):3988. doi: 10.3390/ma17163988 (PMC11356230; doi:10.3390/ma17163988)
Supplement: Supplementary file 1 [file materials-17-03988-s001.zip › materials-3085612-supplementary.pdf]

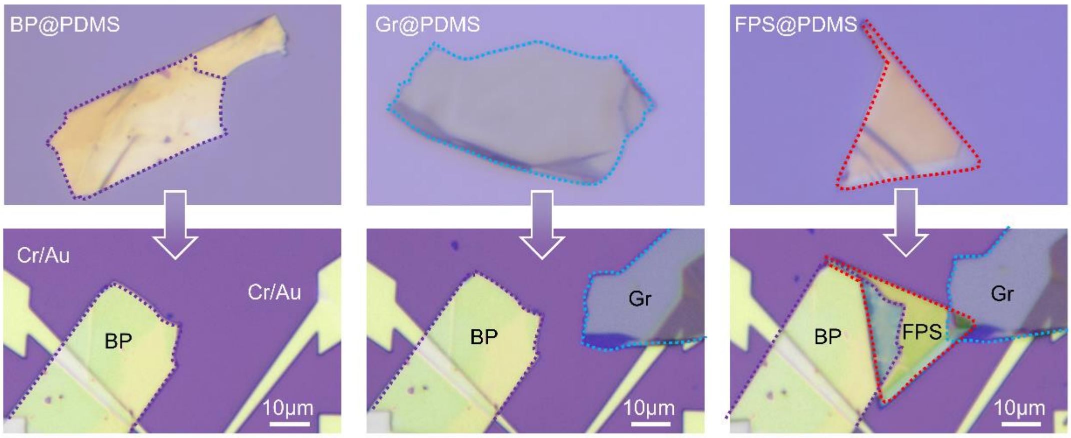

Figure S1. Preparation flow chart of BP-FPS-Gr vdW heterostructure. From left to right, upper panel: optical microscopic images of BP, Gr and FPS nanoflakes attached on PDMS stamps, respectively. Lower panel: corresponding optical microscopic images of different nanoflakes transferred onto the prepatterned Cr/Au electrodes and Si/SiO_2 substrates step by step.

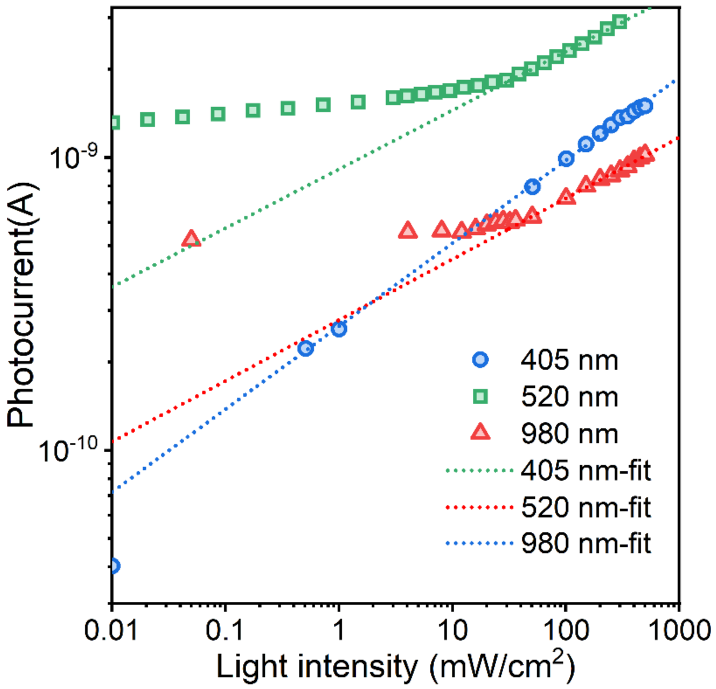

Figure S2. Incident power dependent photocurrents generated by 405, 520, and 980 nm laser excitation.

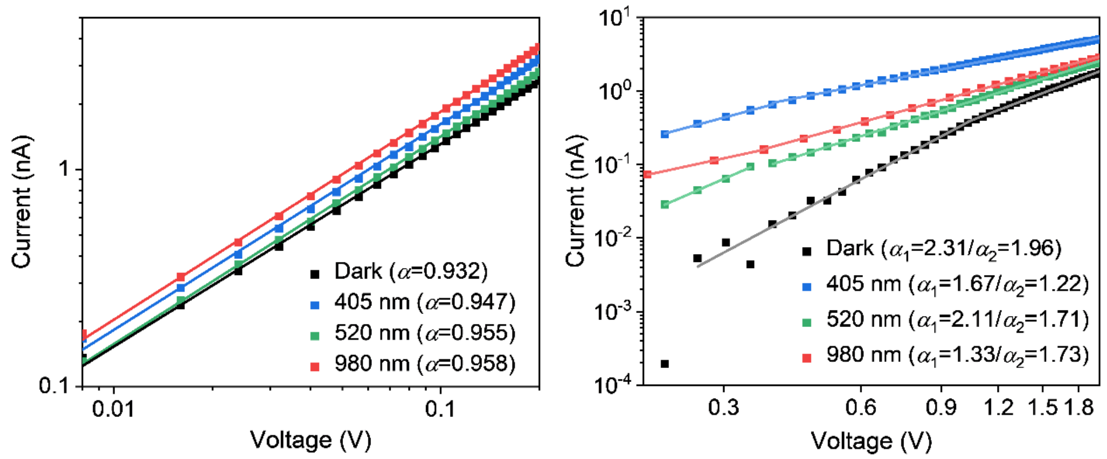

Figure S3. Double logarithmic plot fitting of *I-V* characteristics before (left) and after (right) interface fusion.

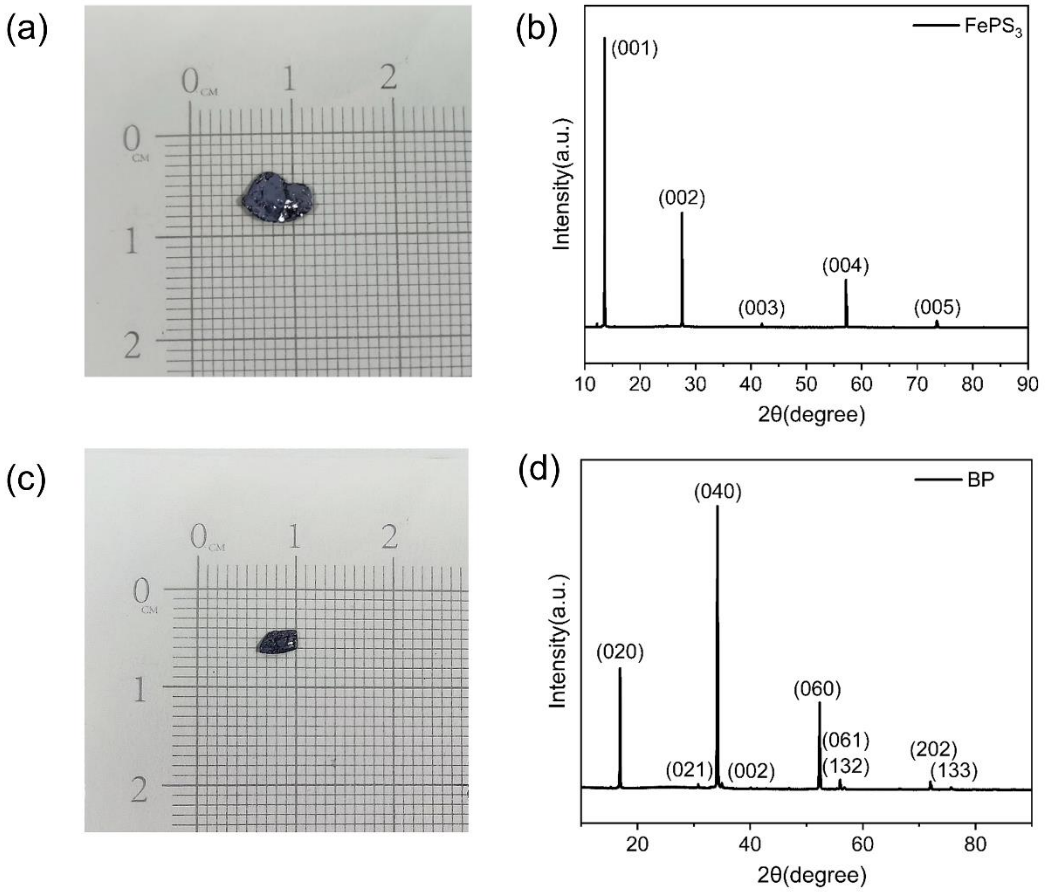

Figure S4. (**a**,**c**) Digital photographs and (**b**,**d**) indexed XRD patterns of $FePS_3$ and BP single crystals.

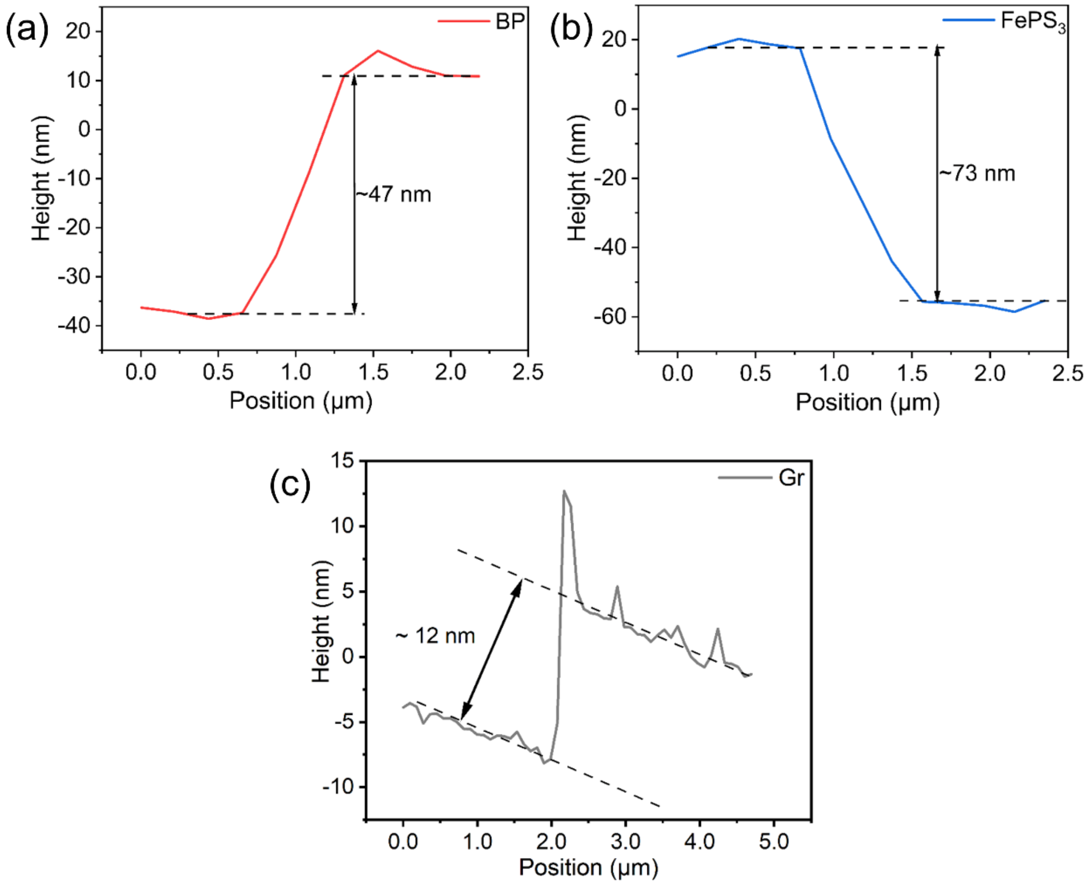

Figure S5. AFM height profiles of micromechanically (**a**) BP, (**b**) $FePS_3$, and (**c**) graphite nanoflakes.

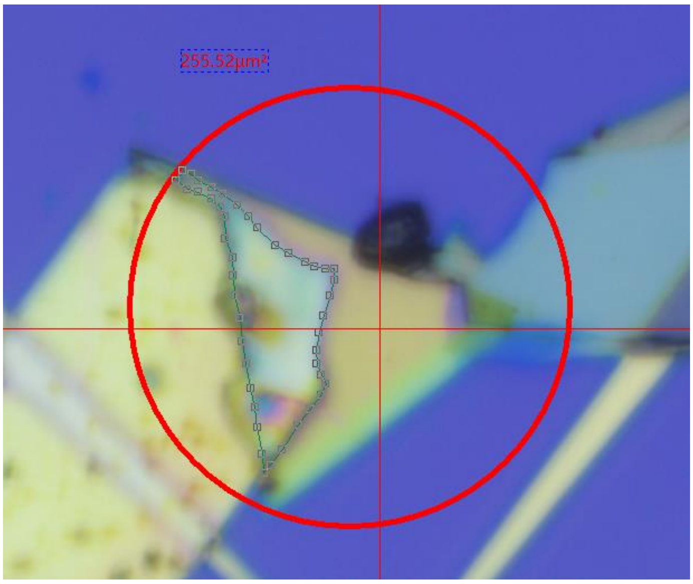

Figure S6. The diameter (55 μm) of the laser beam (red circle) and the overlapped area (255.52 μm^2) of BP-FPS heterostructure.

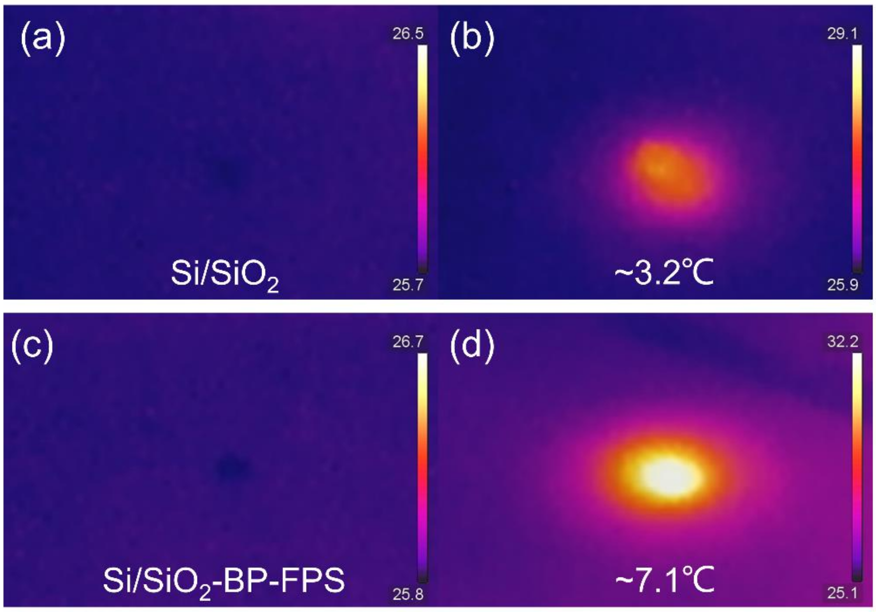

Figure S7. Photothermal imaging pictures of (**a**,**b**) Si/SiO_2 and (**c**,**d**) Si/SiO_2-BP-FPS substrates (**a**,**c**) before and (**b**,**d**) after laser illumination (P_{in}~100 mW/cm^2).

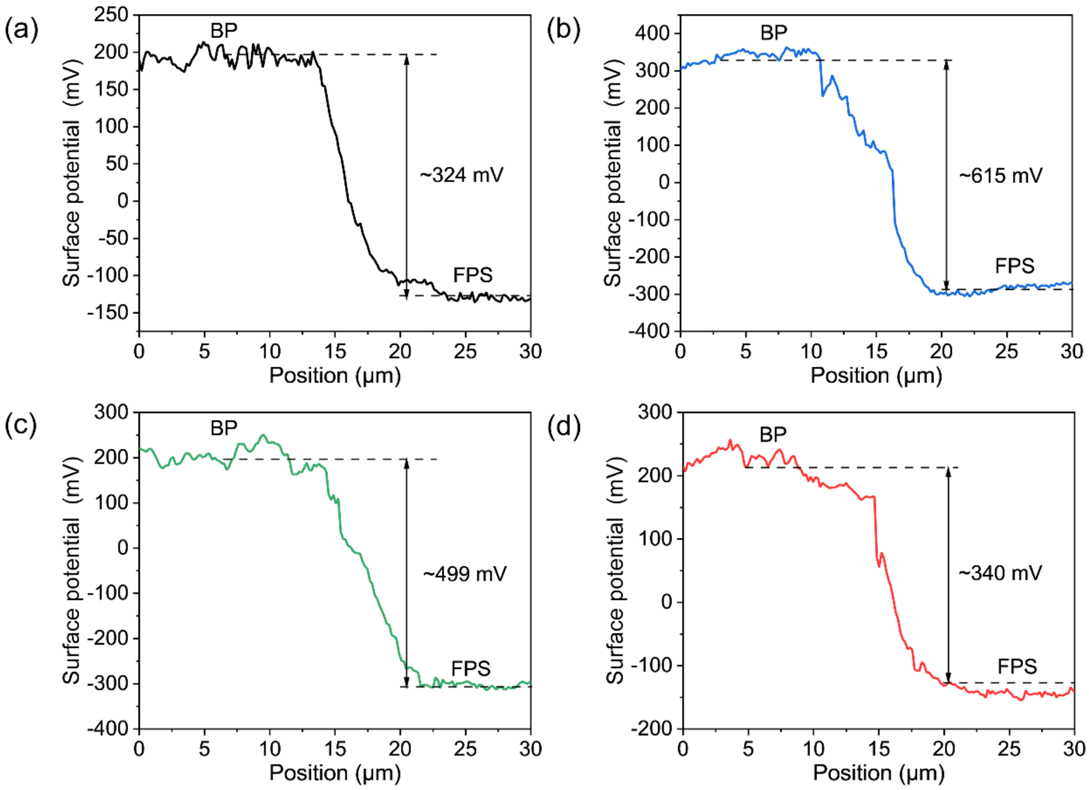

Figure S8. KPFM line-scan profiles of BP-FPS region under (**a**) dark condition, (**b**) 405 nm, (**c**) 520 nm, and (**d**) 980 nm laser irradiation.

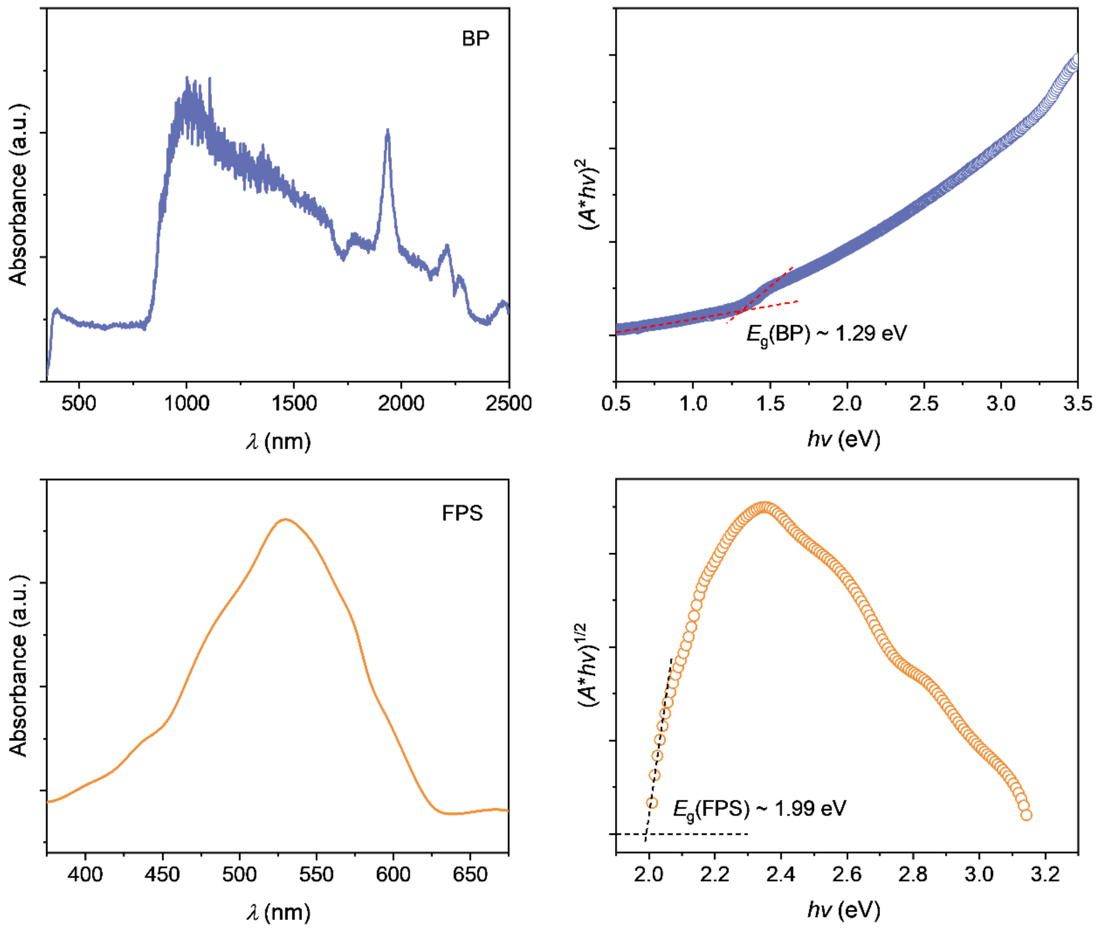

Figure S9. Absorption spectra and corresponding Tauc plots for determining optical bandgap of BP and FPS nanoflakes. We can get the optical E_g of BP and FPS flakes by the linear extrapolation of $[(A*hv)^n\text{-}hv]$ plots, where n=2 for direct bandgap (BP) and n=1/2 for indirect bandgap (FPS).

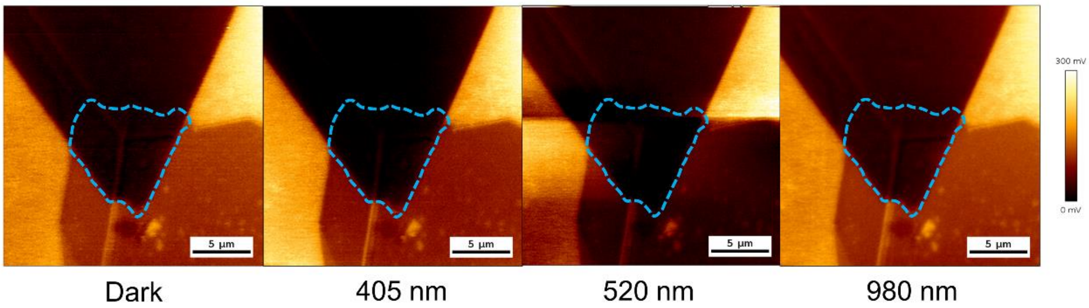

Figure S10. KPFM mapping images for the FPS-Gr heterostructure region under dark and different wavelength excitation conditions.
